# Carpal tunnel syndrome caused by lipoma: a case report

**DOI:** 10.11604/pamj.2015.22.51.7650

**Published:** 2015-09-18

**Authors:** Mohamed Ali Sbai, Sofien Benzarti, Hichem Msek, Monia Boussen, Adel Khorbi

**Affiliations:** 1 Orthopedic Surgery and Trauma Department, MT Maamouri Hospital, Nabeul, Tunisia; 2 Emergency Department, Mongi Slim Hospital, La Marsa, Tunisia

**Keywords:** Lipoma, carpal tunnel syndrome, median nerve, nerve compression

## Abstract

Lipoma is a relatively frequent, benign soft-tissue tumor rarely located in the hand. A lipoma of the hand causing a carpal tunnel syndrome by compression of the median nerve is exceptional. We report the case of a 70-year-old female presenting with a carpal tunnel syndrome. A compression of the median nerve by a lipoma was discovered during surgery. Transverse carpal ligament release with lipoma excision and neurolysis of the median nerve were performed. Histopathological study of the resected mass was consistent with a lipoma. Two-month postoperatively, the patient recovered full hand function with entire disappearance of acroparesthesia. Carpal tunnel syndrome caused by space occupying lesions is rare. Diagnosis is difficult, usually based on the clinical study, electrophysiology and magnetic resonance imaging (MRI). Transverse carpal ligament release and excision of lipoma provides excellent functional recovery.

## Introduction

Soft tissue lipoma is the most common benign tumor found in limbs, although the occurrence of lipoma in the hand remains rare, between 1 and 3.8% of benign tumors in the hand [1]. Carpal tunnel syndrome (CTS) is the most frequent peripheral compression neuropathy, although a CTS caused by a space occupying lesion is rare and causes more complications than idiopathic CTS. We report an exceptional case of a 70-year-old female presenting with a CTS due to a compression of the median nerve by a lipoma.

## Patient and observation

**Figure 1:**
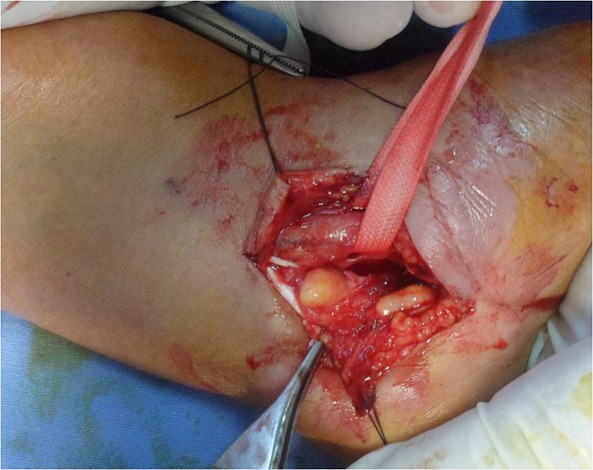
Intraoperative view showing the median nerve, it is flattened, suffering and compressed by the mass

**Figure 2:**
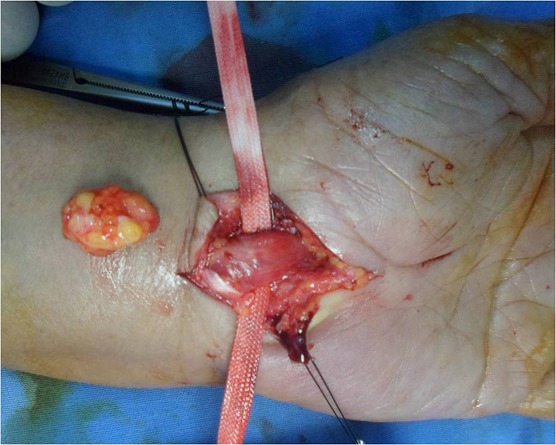
The tumor was carefully removed measuring 2.5 x 1.5 x 1 cm

A 70-year-old left-handed woman, without any significant pathological history presented with numbness and tingling sensation in the left hand affecting thumb, index and middle finger, with a decreased grip strength, appeared 6 months ago. Interrogation revealed a progressive evolution of nocturnal acroparesthesia over several months. Physical examination did not reveal any soft tissue mass on palpation, with a normal range of motion of the wrist (extension 70^°^, flexion 75^°^). There was a decrease in sensitivity in the territory of the median nerve as compared with the contralateral hand without evidence of motricity impairment. The Tinel′s sign was positive at the wrist. Electophysiological study confirmed the diagnosis of CTS. Standard X-rays did not found any abnormalities. Surgery was performed under locoregional anesthesia with axillary block and with tourniquet controlled hemostasis maintained during the procedure. The patient was arranged in a dorsal decubitus position. A palmar conventional approach of the carpal tunnel was chosen. Transverse carpal ligament was incised. Intra-operatively, a lipomatous mass occupying the carpal tunnel space was discovered. The median nerve was carefully identified, It was flattened, suffering and compressed by the mass (Figure 1). The tumor was carefully removed measuring 2.5 x 1.5 x 1 cm (Figure 2) and neurolysis of the median nerve was performed. Histopathological study of the resected mass was consistent with a lipoma with no evidence of malignancy. No immobilization was associated. At the clinical review of two months postoperatively, the patient recovered full hand function (complete mobility of the fingers and normal grip strength) with entire disappearance of acroparesthesia.

## Discussion

Lipomas are tumors developed from mature adipocyte cells, usually encapsulated and sometimes infiltrating [2], they usually grow slowly which explains their often large size at diagnosis, especially in deep locations. Lipoma is often painless and usually discovered by palpation of a soft, regular and movable mass when it is superficial. The localization in the hand remains rare: 5% of cases, and depending on its location it may cause, interosseous nerve compression in forearm [3], a CTS, a compression of the ulnar nerve in the Gyon tunnel [4] or even digital nerves compression [5]. The vascular compression with distal ischemia was not reported in the literature. The nerve compression signs are not correlated to the size of the tumor. In the consulted series, compression of the median nerve was sometimes observed for small tumors. The nerve compression, in these cases, has been explained by the intraoperative discovery of an adherent tumor [6]. Idiopathic CTS is usually known to occur bilaterally. 66% of patients with unilateral CTS show anomalies in nerve conduction test results on contralateral side [7]. Other reasons for CTS should be suspected, besides idiopathic CTS, when patient's symptoms and nerve conduction test show unilateral anomalies. Lipoma is a very rare cause of CTS, only few cases were reported in the literature [5, 7–9], out of 779 patients with CTS, Chen found 3 lipomas in 23 space occupying lesions [8]. In a serie of 568 cases of CTS, Ho Jung Kang did not report any cases of lipoma [9]. CTS secondary to space occupying lesions has an atypical expression, ultrasound should be requested, it is effective for exploration of any mass in the hand, besides its low cost and availability.

MRI is a useful tool for the diagnosis of soft tissue tumors, because of its high sensitivity, it specifies the nature of the lesion. It also allows a preoperative planning by studying the dimensions of the tumor, its local extension and its relationship with the neuro-vascular elements. Usually, lipomas appear as an homogeneous mass, with a sharp border and spontaneous T1 and T2 hyper signals with reduction of signal on fat suppressed sequences. In some cases, the image contains fibrous septa or calcifications. After gadolinium injection, the fibrous septa signal moderately raises, but fat keeps the same signal [10]. In a series of 134 tumors and pseudo tumor of the wrist and hand, lipoma suspected preoperatively on MRI was confirmed by histology in 94% of the cases [11]. The diagnosis of liposarcoma is rare, however it cannot be dismissed. Any soft tissue mass larger than 5 cm should be regarded as malignant until proved otherwise, only the Histopathological study of the resected mass allows to eliminate it [12]. In the hand, complete resection of the lipoma is the treatment choice that allows liberation of the compressed nerve. Conventional open approach is recommended with contraindication for endoscopic procedures. Dissection and identification of neuro-vascular elements must be careful to avoid iatrogenic injuries. Transverse carpal ligament release and resection of lipoma usually provides excellent results. Postoperatively, patients quickly recover full hand function with entire disappearance of pain and acroparesthesia. Local recurrences are exceptional.

## Conclusion

Lipoma is uncommon in hand and wrist, even rarer as a cause of secondary CTS, however it should be suspected. A thorough clinical examination, Functional exploration with electromyography, morphological study with MRI are necessary. Conventional open approach is recommended with contraindication for endoscopic procedures. Complete and successful surgical resection provides excellent functional recovery. Preoperative assessment and patient information are necessary for proper management. Histology after complete removal of the tumor remains necessary to confirm the diagnosis and eliminate malignant tumor, in particular a liposarcoma.
